# Evaluation of nano glass as a partial cement replacement on the fresh mechanical durability thermal and microstructural properties of cement paste

**DOI:** 10.1038/s41598-026-37244-0

**Published:** 2026-01-27

**Authors:** Sherzad Mohammed Ali, Shwan Abdullah Mohammed, Adnan Ahmed Juma, Hersh F. Mahmood, Bzhar Muheddin Mohammed, Dler Ali Ahmad, Soran Abdrahman Ahmad

**Affiliations:** 1 Water Resource Department, Collage of Engineering, University of Sulaimani, Sulaimaniah, Kurdistan Region Iraq; 2 Sulaimani Technical Institute, Sulaimani Polytechnic University, Sulaimani, Kurdistan Region Iraq; 3 Civil Engineering Department, University of Garmian, Kalar, Kurdistan Region Iraq; 4 Civil Engineering Department, University of Halabja, Halabja, Kurdistan Region Iraq; 5 Civil Engineering Department, College of Engineering, University of Sulaimani, Kurdistan, Iraq

**Keywords:** Waste glass, Paste, Mechanical properties, Pozzolanic reaction, XRD, Thermal conductivity, Engineering, Materials science, Nanoscience and technology

## Abstract

Using nano materials in the construction sector is one of the new directions as the most effective method for improving the quality of produced materials, especially in concrete and mortar that used as a partial replacement of available material or admixture that is considered. These materials, which are produced in nano size, have been investigated in previous research with micro size may also be with higher size. This study investigates the effects of nano glass powder as a partial replacement for cement on the fresh, mechanical, and durability properties of cement paste. Nano glass was incorporated at varying replacement levels (0–50%) and evaluated through a series of experimental tests, including flowability, compressive and flexural strength, acid resistance, thermal conductivity, fire resistance, water absorption, density, permeable voids, drying shrinkage, X-ray diffraction (XRD), and scanning electron microscopy (SEM). The results reveal that increasing nano glass content reduces flow values, indicating decreased workability due to higher water demand. Compressive strength (C.S) improved at early (28-day) and later (56-day) ages, peaking at 10% replacement, while flexural strength peaked at 15%, showing an optimal range for mechanical enhancement. Durability tests showed improved acid resistance and reduced thermal conductivity at moderate replacement levels, confirming enhanced long-term performance. Fire resistance analysis indicated improved strength retention and reduced weight loss with higher nano glass contents, particularly between 20% and 30%. Water absorption and permeable voids decreased significantly at higher replacement levels, indicating denser and more durable microstructures, although density dropped at 40% replacement. Drying shrinkage was minimized at 10%, while XRD and SEM analyses confirmed compositional and microstructural improvements due to pozzolanic reactions and densification. Overall, the study demonstrates that nano glass can be effectively used up to 15–20% replacement to enhance strength, durability, and thermal resistance, though excessive amounts may negatively affect workability and structural integrity.

## Introduction

More than thirty percent of the produced energy in the world is used in the construction sector^1,2^, since concrete is one of the materials used whose usage quantity has passed eleven billion tons annually, considered as one of the main users of energy^3,4^. Among the concrete compositions, the cement industry is considered a real threat to the environment since cement production requires the highest amount of energy among concrete compositions for its production, also producing one ton of cement emitting about one ton of carbon dioxide into the air^4,5^. The cement industry is considered responsible for emitting about eight percent of the total carbon dioxide into the air^5–8^. The cement used annually passes four billion tons and is estimated to pass six billion tons by 2050^9,10^. More than three percent of the total energy used in the world is used by the cement production industry^11^.

Many researches has been done to find alternative materials that can be used as a replacement for cement or a partial replacement of cement. As a result, many materials have been found that have pozzolanic properties, such as fly ash, silica fume, ground granulated blast furnace slag, and met kaolin^12–14^. Obtaining pozolanic material from waste sources is considered a more sustainable result since it cleans the environment by reducing cement production and makes the produced material sustainable^15–19^. Using pozzolanic materials with the nano size will be more active compared to the same materials with micro size^20,21^.

One of the solid waste materials that have pozzolanic properties is waste glass^5,22–24^. Glass is non-biodegradable material which make it to need long time in the environment to recycled^25–27^, which is with the increase of the population increase its produced waste quantity due to its high applications in human life^28–33^. The produced quantity of waste glass was about twelve million tons in 2010 in the United States, while it passed thirteen million tons in 2011^34–36^. Generated waste glass passed 0.75 million tons in Iran annually^37–40^, in Turkey, more than one hundred twenty thousand tons of glass annually are put in the landfill^41^. In Taiwan, more than half a million tons will be put in the landfill annually^42^. More than three hundred tons of waste glass are produced and put in the landfill annually in Hong Kong^43–47^. Totally, the produced waste glass that is put in the landfill is about two hundred million tons^48^.

Many researches have been done to use waste glass granular as partial replacement of coarse aggregate in concrete^49,50^, and as fine aggregate in concrete and mortar^51–53^, while many researches have been done to use waste glass powder as partial replacement of cement in concrete, mortar and paste^54–60^.

Elaqra and Rustom^61^ used waste glass powder as a partial replacement of cement in the paste with five different rates (0, 10, 20, 25, and 30) using two different types of cement to find the effect of the cement type on the behavior of glass powder. As a result, it was found that the rate of the glass powder that provides higher performance of the paste changed based on the chemical composition of the cement. Patel et al.^62^, measured the effect of the particle size of using waste glass powder as partial replacement of cement in paste and mortar when brought the waste glass powder with two different size (75 µm, and 63 µm) used them as partial replacement of cement with five different rate (0, 5, 10, 15, and 20%). The obtained result showed that the usage of waste glass powder with finer particles provides a less workable mix, higher performance of strength, and pozzolanic reactions. Jiang et al.^63^, tested the usage of waste glass powder in the geo-polymer paste based fly ash to find its effect on the workability, setting time, bond strength, and mechanical performance of the paste, the result showed the decrease in the setting time, increase in the workability while with the usage of 20% of waste glass powder as partial replacement of fly ash the highest bond strength and mechanical performance will obtained. Nahi et al.^64^ investigate the usage of glass powder as a partial replacement of cement in the paste and mortar with five different rates (0, 10, 25, 35, and 60%), to find the chemical performance of the paste and the mortar. The obtained result showed that the reaction between silicate oxide in waste glass powder and calcium hydroxide in the hydration reaction will produce calcium silicate hydroxide which fills the voids and increases the density, mechanical performance of the paste. Liang et al.^65^ used waste glass powder as a partial replacement of met kaolin in the geo-polymer paste to find its effect on the mechanical performance of the paste, which found that with the usage of waste glass powder by 20% as partial replacement of met kaolin, the highest mechanical performance of the paste was obtained. Arowojolu et al.^66^, investigate the usage of nano glass as partial replacement of cement with class F-fly ash by three different rate which include 0, 12.5, 25% when the water to cement ratio was constant to investigate its effect with fly ash on the fresh, mechanical, and durability of concrete, the obtained result showed that with the increase of the nano glass usage decrease the flow ability value, while using 12.5% of nano glass with 12.5% of fly ash decrease the mechanical properties of concrete, but the value of alkali silica reaction still remain the accepted range for normal situation. Onaizi et al.^67^ used nano glass as an additive in concrete with three different rates, including 0, 5, 10% in two groups of mix, when the first group contained fly ash by 50% as cement replacement with a 0.45 water to cement ratio. The second group also contains 50% of fly ash as cement replacement with a 0.5 water-to-cement ratio. As a result, it was found that the addition of nano glass decreases the workability of the mix, but the performance of the mix will increase, especially with the usage of 5% of nano glass. This article deals with the investigation of using nano waste glass as a partial replacement of cement with eight different rates in the paste to evaluate its effect on the fresh, mechanical durability, thermal conductivity, fire resistance, water absorption, and dry shrinkage of the paste, and showing the real behavior of the nano glass powder.

## Research significant

This article deal with the usage of the nano waste glass as partial replacement of cement in paste with different rate to measure (i) its effect on the fresh, mechanical, durability, thermal conductivity, fire resistance, water absorption and dry shrinkage of the paste, (ii) also to select optimum rate of the nano glass powder that provide optimum effect on the properties of the paste, with (iii) measuring performance and real behavior of nano glass with cement using x-ray diffraction (XRD) and scanning electron microscope test (SEM).

## Methodology

**Fig. 1:**
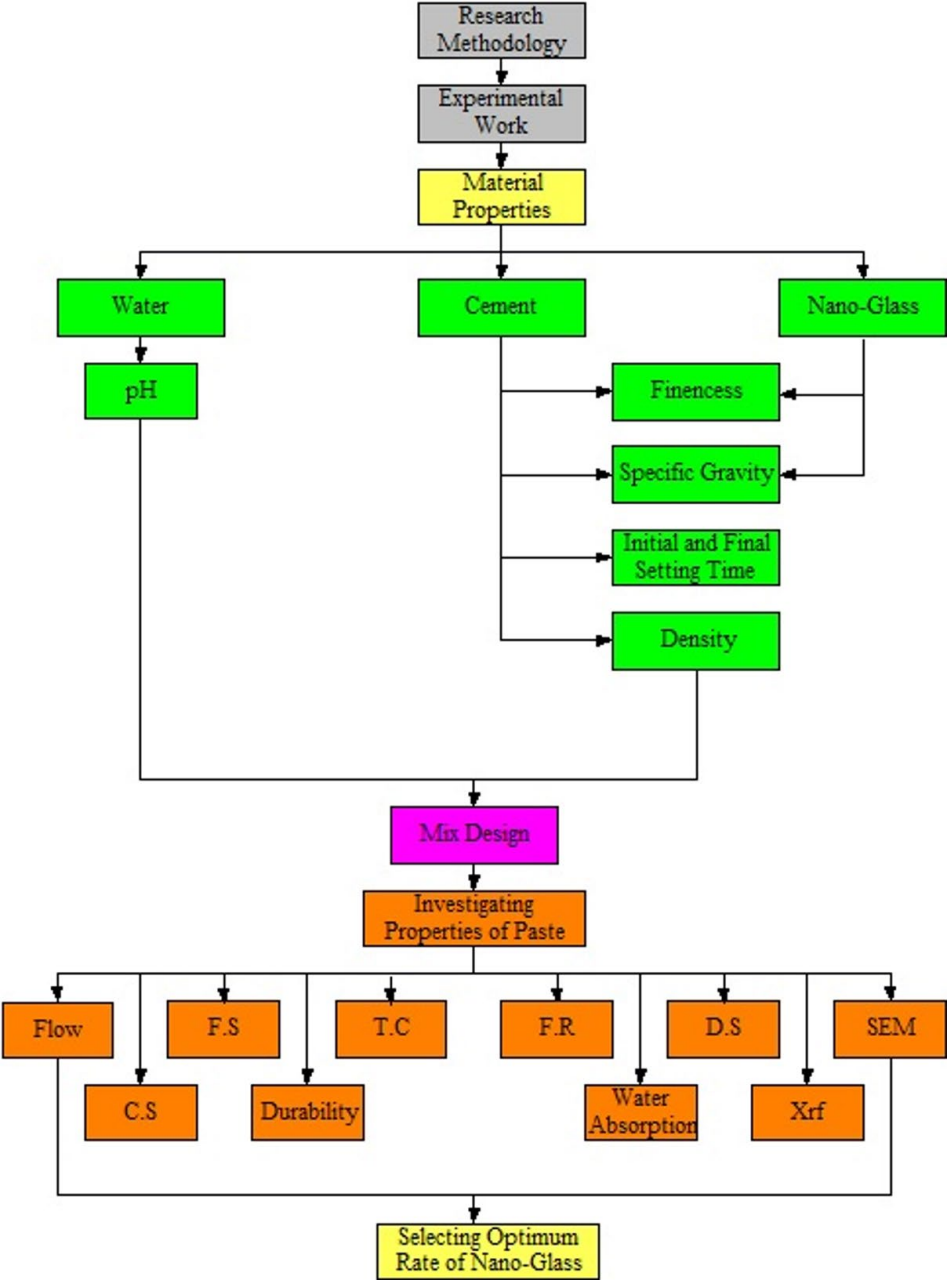
Research methodology.

This article deals with the usage of nano glass as partial replacement of cement in paste with different rate including 0, 5, 10, 20, 30, 40 and 50% to find their effect on the fresh, mechanical, durability, and thermal conductivity properties of paste for that the properties of used materials have been investigated, followed by designing the mix of the paste and the modified pastes, finally the required samples for the investigated properties (flow, C.S, flexural strength, durability, thermal conductivity, fire resistance, water absorption and dry shrinkage), have been prepared and cured. After the selected time of curing, the experimental tests have been done as explained in Fig. 1.

## Experimental work

In this part, at the first step, the properties of the used materials have been investigated, followed by the mix design for the control pastes and the modified pastes. Finally, the required samples for the investigated properties of the paste have been prepared.

### Used materials

The mixed composition of the paste is water and cement, since the target of this article is to modify the cement paste with different rates of nano glass, it will be necessary to investigate the properties of nano glass also.

#### Water

The used water has been passed through a pH test to find the purity of this water to avoid the usage of water that can react chemically with other mixed components for Based on the given procedure in ASTM, C1293^68^, the pH value of the used water was 7.4, which means acceptable.

#### Cement

**Fig. 2:**
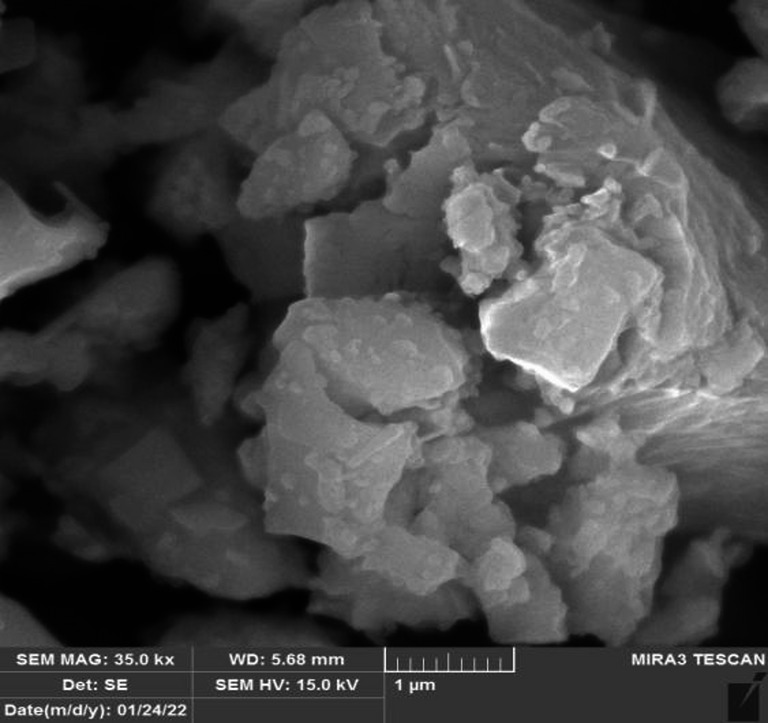
Particle texture of used cement using SEM.

The used cement was from Delta Cement Company in Sulaimaniyah city. The fineness value was 3539 square centimeter (cm^2^/gram), which was measured based on ASTM, C115^69^, while its density is 1442 kg/m^3^ based on ASTM, C188^70^. The initial and final setting times of the delta cement were 144 and 193 min, which were determined according to the given procedure in ASTM C191^71^. The specific gravity was 3.14^70^. All the investigated properties have been in the limitation to Portland cement as selected in ASTM, C150^72^. The used cement has the chemical composition as expressed in Table 1 and particle texture as shown in Fig. 2.

**Table 1:** Chemical composition of cement.

Composition name	Composition percentage (%)
Silicon dioxide	19.12
Aluminum oxide	4.53
Iron(III) oxide	4.55
Calcium oxide	62.52
Magnesium oxide	3.75
Sulfur trioxide	2.42
Potassium oxide	0.47
Sodium oxide	0.12
Carbon dioxide	2.53

#### Nano-glass

**Fig. 3:**
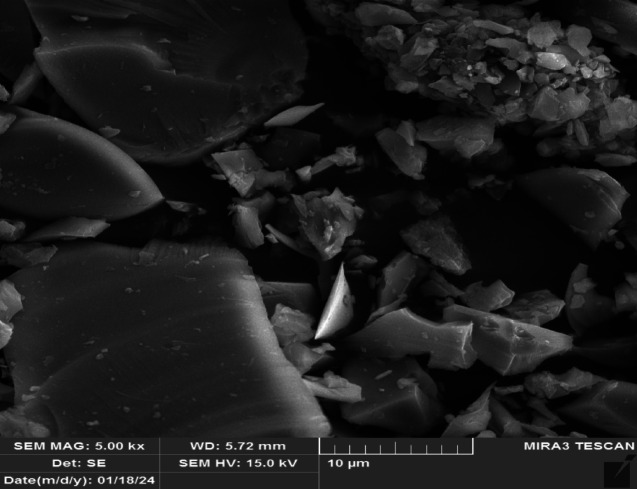
Particle texture of used nano glass using SEM.

The used nano waste glass in this article has been obtained from waste bottles and crushed to the nano size as shown in Fig. 3. The chemical composition of the nano glass has been expressed in the Table 2. The fineness value was 6775 cm^2^/gram which measured based on ASTM, C115^69^, the specific gravity was 2.88^70^.

**Table 2:** Chemical compositions of nano glass.

Composition name	Composition percentage (%)
Silicon dioxide	58.24
Aluminum oxide	2.62
Iron(III) oxide	1.12
Calcium oxide	2.48
Magnesium oxide	0.86
Sulfur trioxide	0.53
Potassium oxide	6.77
Sodium oxide	6.69

### Used mix for prepared mixes

Based on the conditions as described in ASTM, C305^73^, the mix compositions are as expressed in the Table 3 have been mixed to fill prepared molds for the required samples.

**Table 3:** Mix compositions based on the different used rate of nano glass.

Mix name	Replacement rate (%)	w/c	Water (gram)	Cement (gram)	Nano-glass (gram)
Mix-1	0	0.3	195	650	0
Mix-2	5	0.3	195	617.5	29.8
Mix-3	10	0.3	195	585	59.6
Mix-4	15	0.3	195	552.5	89.4
Mix-5	20	0.3	195	520	119.25
Mix-6	30	0.3	195	455	178.9
Mix-7	40	0.3	195	390	238.5
Mix-8	50	0.3	195	325	298

### Investigated properties

**Fig. 4:**
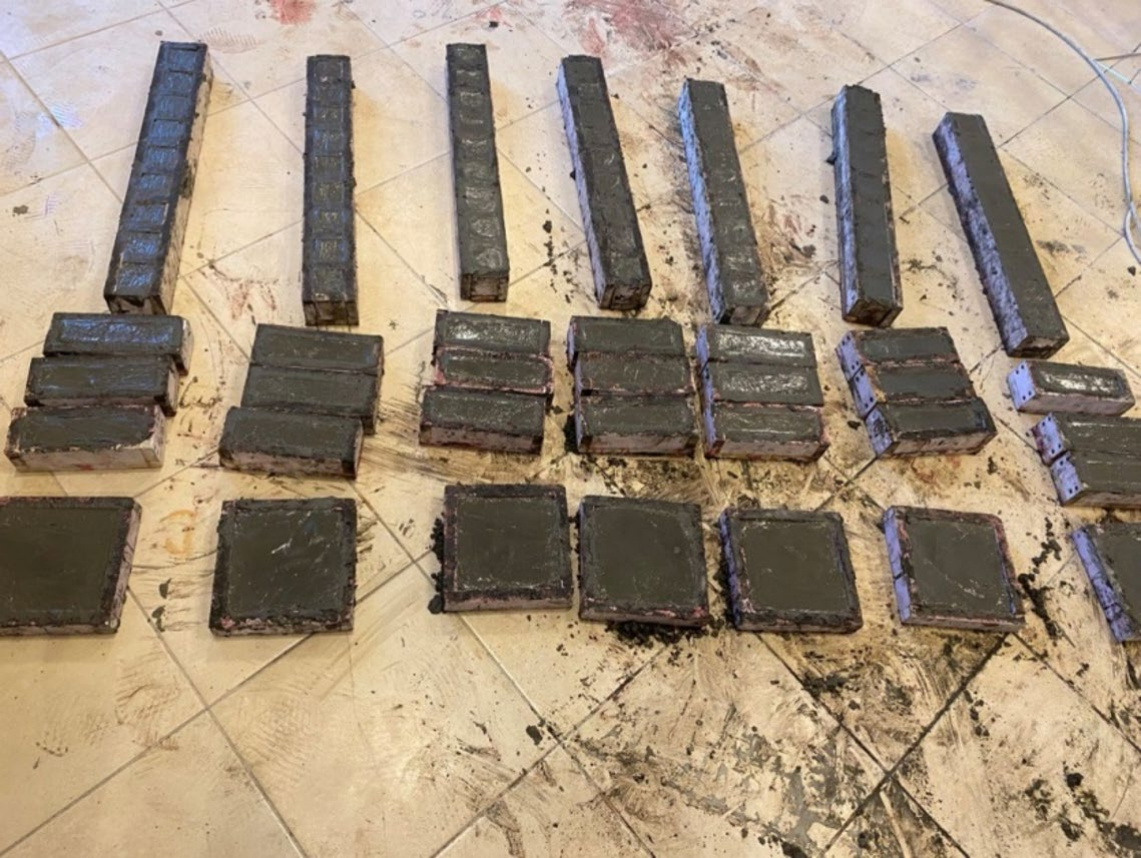
Prepared samples for the investigated properties.

After finding the normal consistency of the cement paste, the mix compositions have been prepared and mixed to prepare the samples that are required for the investigated properties of the paste, as shown in Fig. 4. The investigated properties and their measuring state are as described in the following sub-sections:

#### Flow

**Fig. 5:**
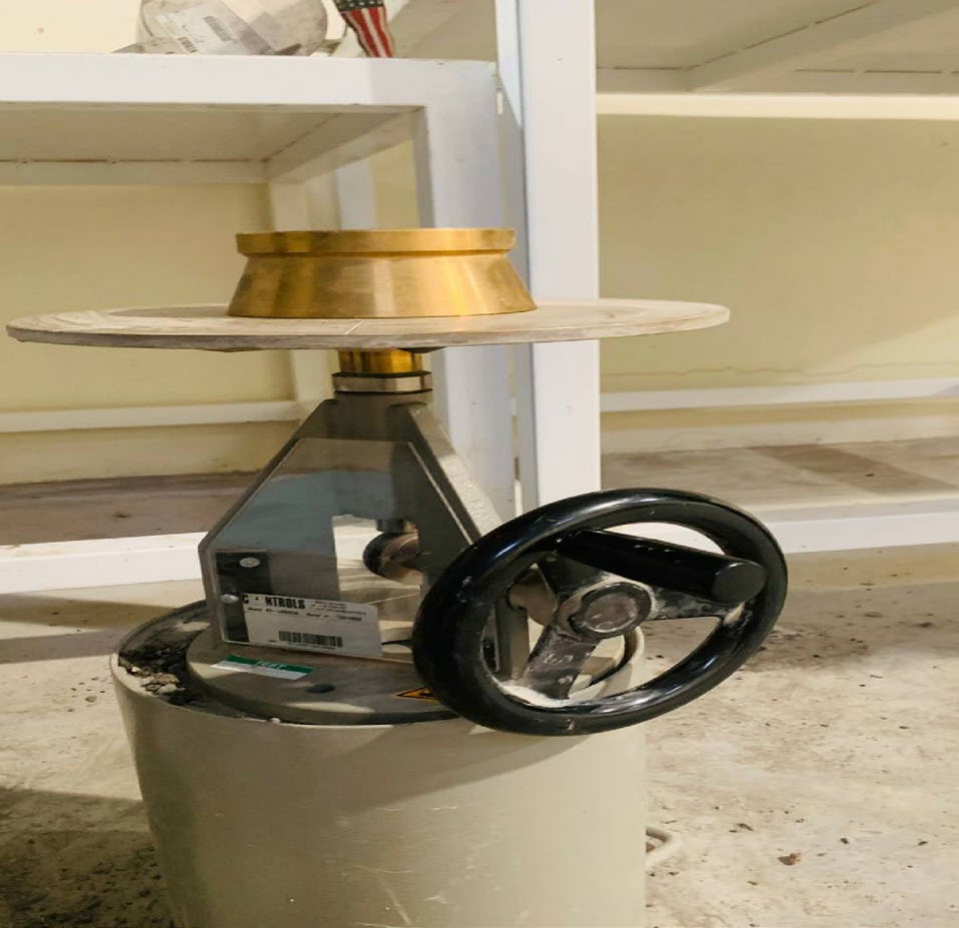
Used flow table in the flow ability test.

Flowability of the paste is the property that expresses the degree of workability of the prepared mix in the fresh state, which is measured according to the procedure explained in detail in ASTM, C1437^74^, by using a flow table that has detailed dimensions as explained in Fig. 5 and ASTM, C230^75^.

The given mold will be put in the center of the flow table and filled with the prepared paste in two layers, and each layer must be tamped twenty times. After filling the top layer, tamping the process, and removing extra paste from the top of the mold, remove the mold and rotate the table twenty-five times in fifteen seconds; after that, measure the diameter of flow in four directions to find the flow as in Eq. (1).

1 $$Flow = \frac{Average\;of\;four\;diameters\;fo\;flow - internal\;bas\;diameter\;of\;mould}{{internal\;base\;diameter }}*100$$

#### Compressive strength

**Fig. 6:**
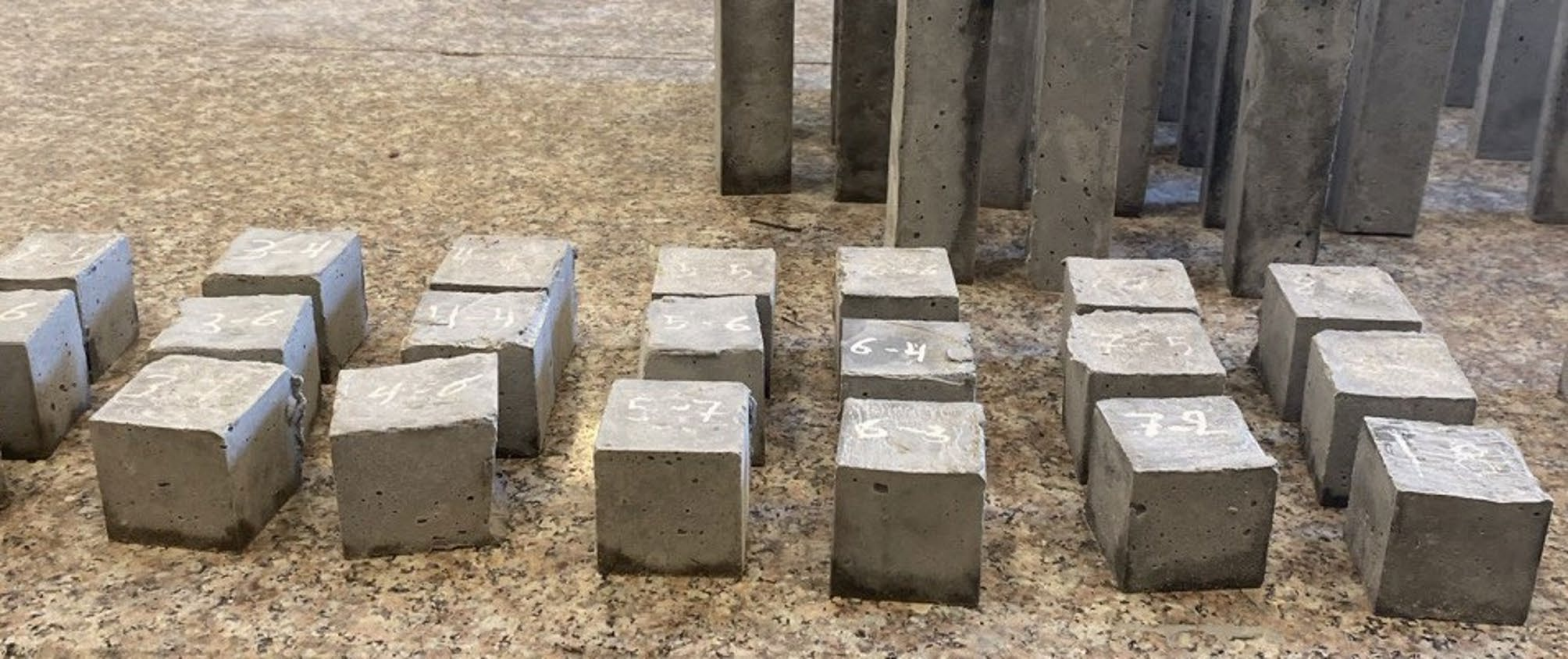
Prepared compressive strength samples.

After the mixing of components, to obtain the effect of adding different rates of nano glass to the paste on the C.S of the paste, for each mix, nine cubes with 5 cubic centimeter have been prepared and cured as shown in Fig. 6. Three cubes have been tested under compressive machine load with a load rate of 900 to 1800 N/s as provided in ASTM, C109^76^ after twenty-eight days of curing time. After breaking the prepared samples at the selected curing time, the C.S will be found for each mix as the average of the C.S of three samples, satisfying that the C.S of each sample in the same mix does not differ by more than 8.7% from the average value.

#### Flexural strength

**Fig. 7:**
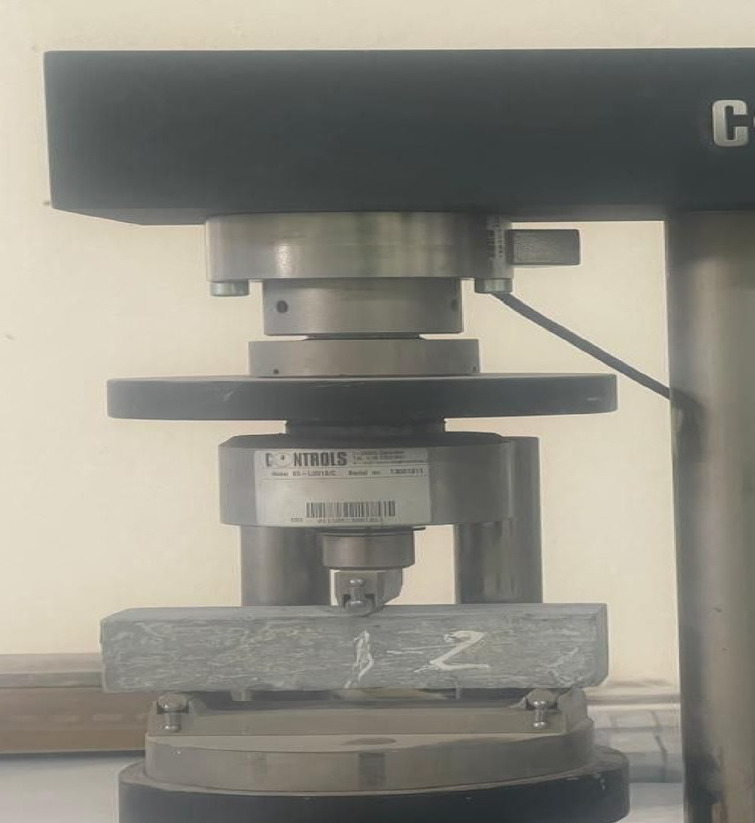
Flexural strength samples.

Flexural strength is one of the mechanical properties that shows the ability of the paste to withstand the bending load. The samples for this test have been prepared with dimensions of 4 × 4 × 16 cm as shown in Fig. 7 and loaded with 2640 ± 110 N for each minute which mean 44 ± 1.8 N/Second after curing time based on the detailed specification in ASTM, C348^77^.

#### Durability

**Fig. 8:**
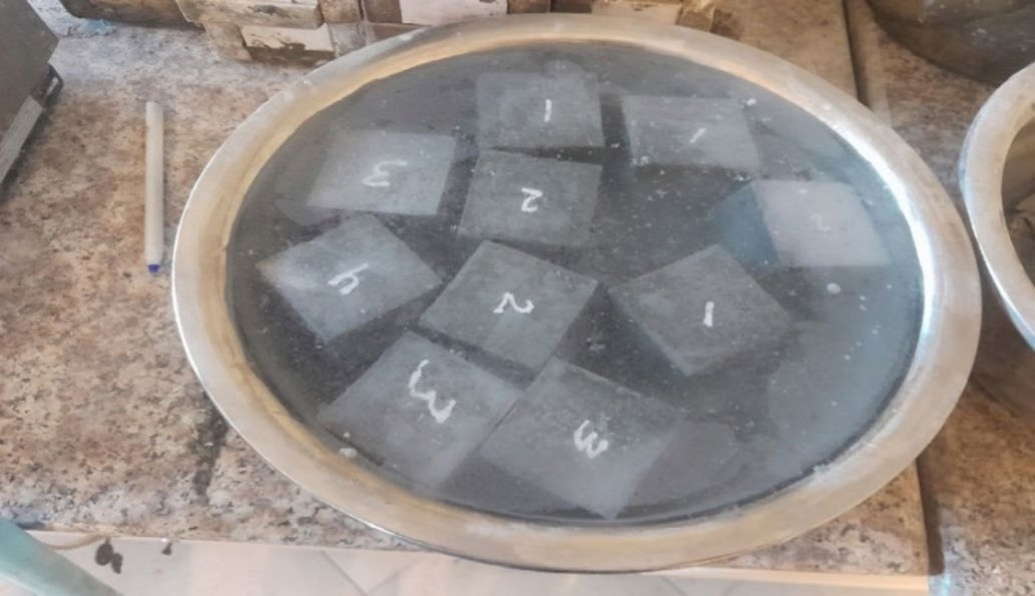
Paste samples inside the curing bath for durability.

In this step, six cubes for each mix remain. After 28 days of curing, three cubes will remain in the water bath for up to 56 days as the total time of curing, while the other three cubes will be taken out after the first 28 days and be cleaned and then put in the bath, which contains 95% water with 5% H_2_SO_4_ for the second twenty-eight days as in the Fig. 8. The samples will remain in this bath for up to 56 days as the total time of curing. After these processes, the samples, including three in a water bath and the other in an acid bath after 56 days, will be their faces cleaned to remove extra water, and tested with a C.S test machine to measure the effect of the acid on the C.S ability of the samples in the acid bath compared to the other samples that cured in the normal water.

#### Thermal conductivity

**Fig. 9:**
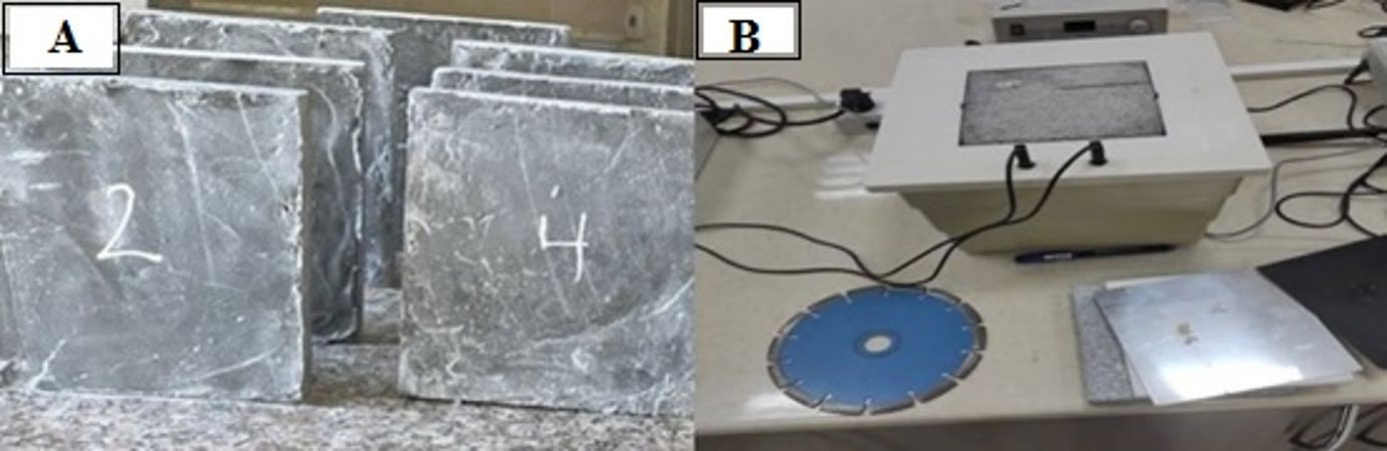
**a** Prepared samples for thermal conductivity test. **b** Sample under thermal conductivity instrument^78^.

Thermal conductivity is the property that shows the ability of passing thermal waves from one face of the material to the other. This test is used to measure the ability of the added material in the mix in reducing the wave transformation through the material. A reduction in thermal conductivity causes an increase in thermal insulation, which reduces the amount of energy that is required for condition control inside the buildings. The used samples in this test are 15 × 15 × 2 cm, as shown in Fig. 9a. The idea of the test is to put the sensor at the top of the prepared sample with another sensor at the bottom of the prepared sample to measure the amount of heat that is put at the first face and compare it to the amount of heat that passes from the other face, as shown in Fig. 9b.

#### Fire resistance

**Fig. 10:**
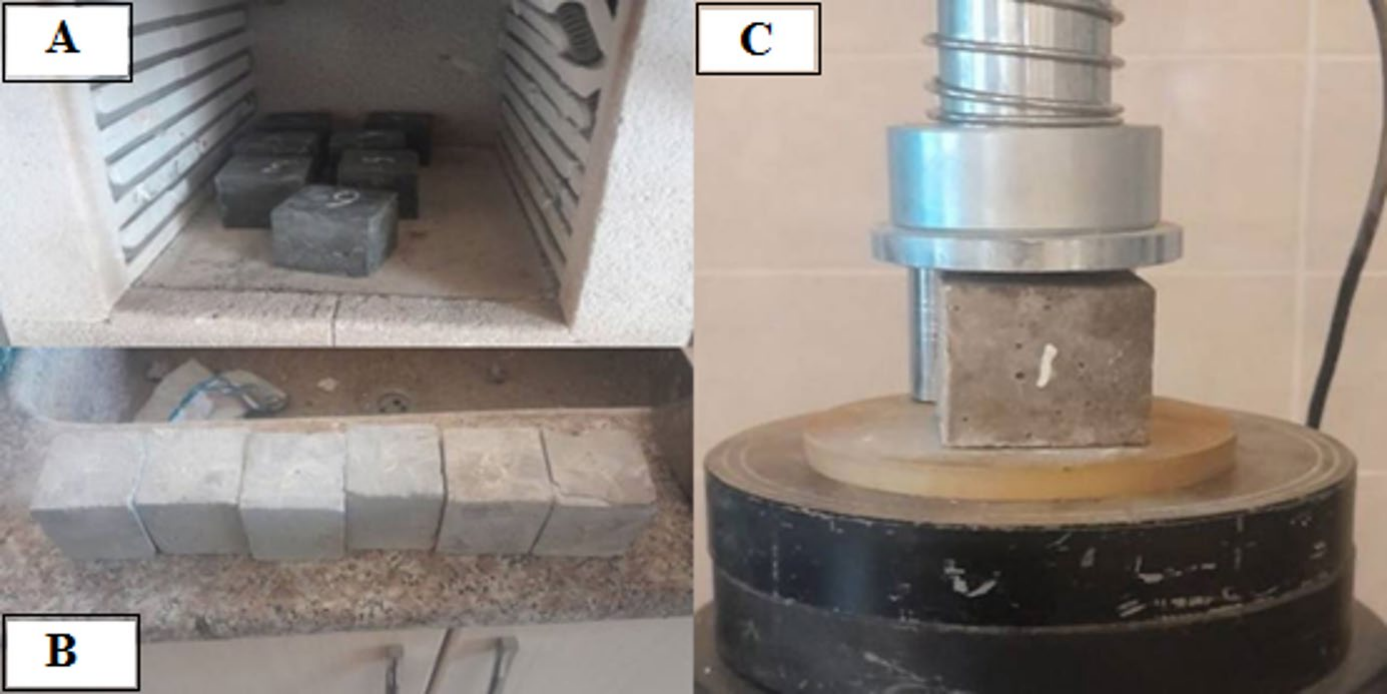
**a** Paste samples for fire resistance test. **b** Paste samples after subjecting to the fire. **c** Fired samples under compressive strength machine.

Fire resistance is the ability of the prepared mix to withstand fire, which is subjected to by investigating the effect of fire on the composition of the mix and the mix’s ability to resist the strength that is applied. Based on the required target and given details in the ASTM, E119^79^, and ISO 834^80^, three cubes for each mix have been prepared and cured for 28 days. Prepared samples have been weighed and put in the oven for one hour after that the degree of the oven has been set on 400 °C, the oven has been turn on, and the temperature started rising up to receive 400 °C after that the samples have been stayed in the oven for one hour in the state of subjecting to 400 °C. after the time passing, the oven has been turn off and the after 15 min of cutting the electric from the oven the door of the oven has been opened and the samples have been taken out to get the temperature degree of the laboratory. They have been weighed to find the amount of weight loss due to the heat-subjecting process. The samples have been broken by a C.S machine, as shown in Fig. 10, to find the effect of the heat on the C.S ability of the pastes and compare the obtained result with samples that were broken after 28 days of curing in a normal situation.

#### Dry shrinkage

**Fig. 11:**
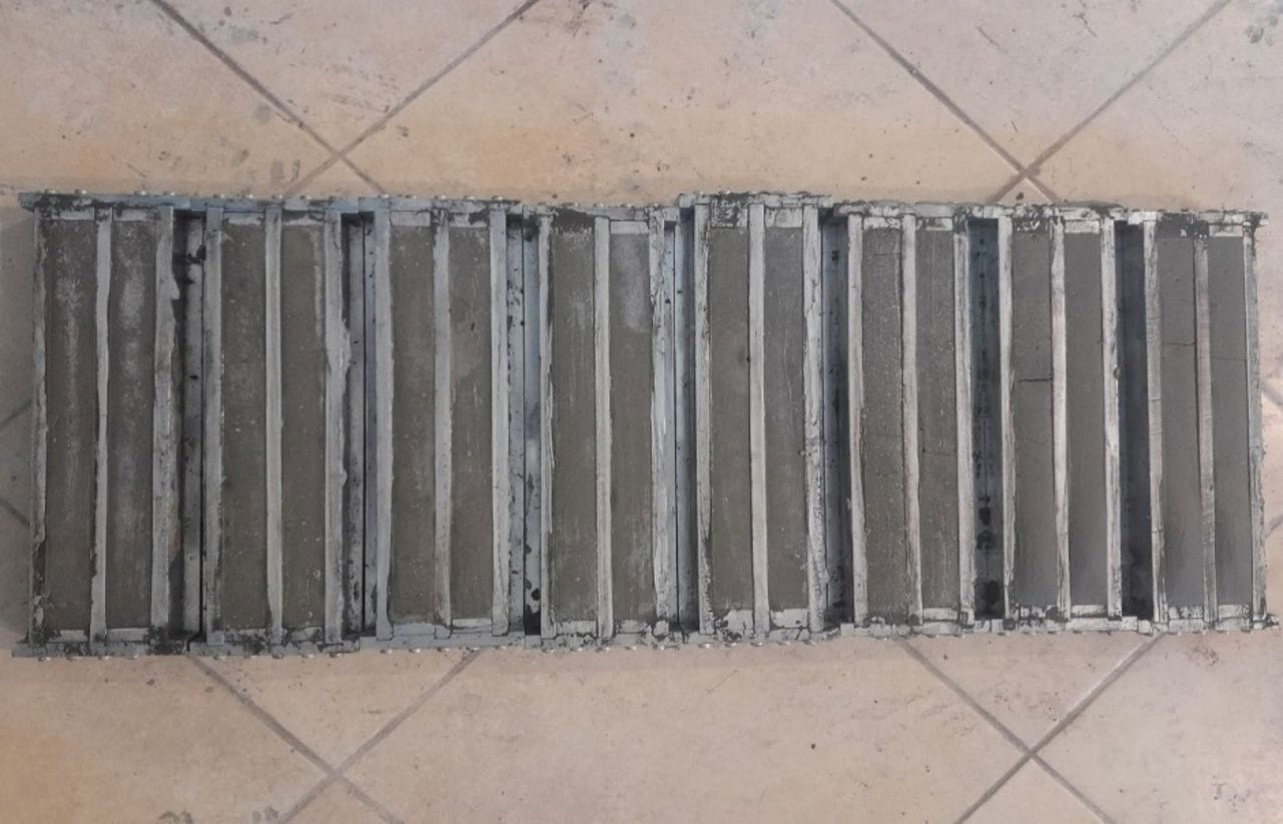
Prepared samples for dry shrinkage test.

**Fig. 12:**
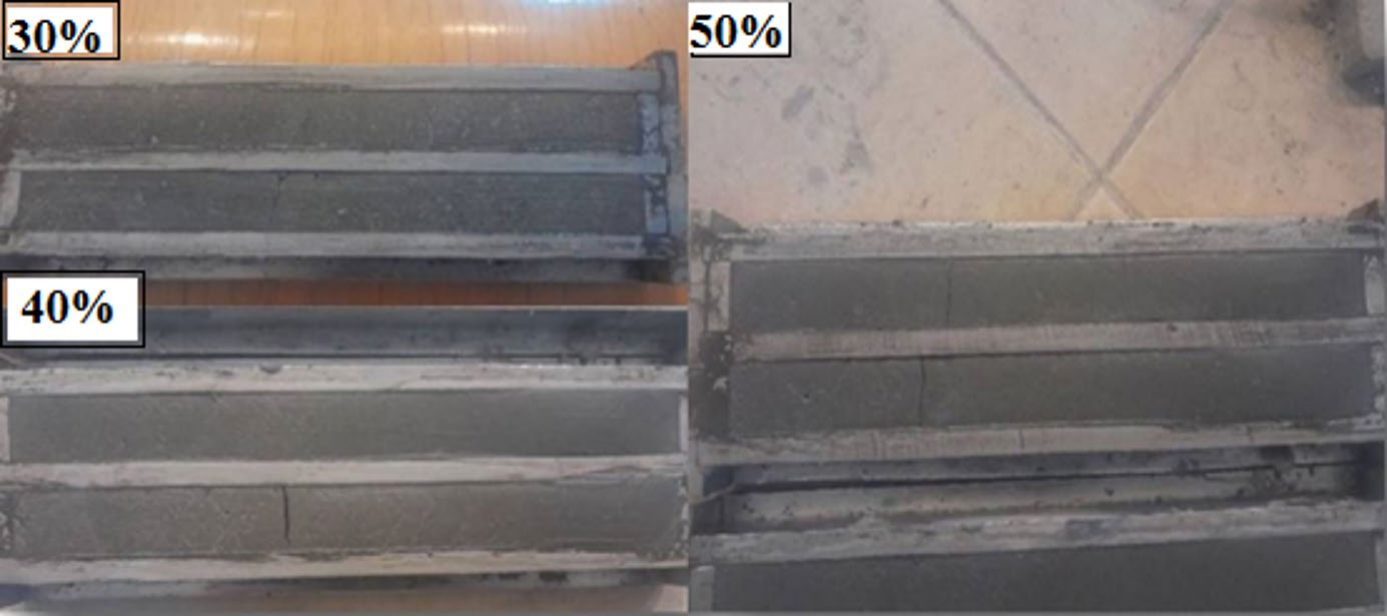
Dry shrinkage sample modified with 30, 40, and 50% of nano glass.

**Fig. 13:**
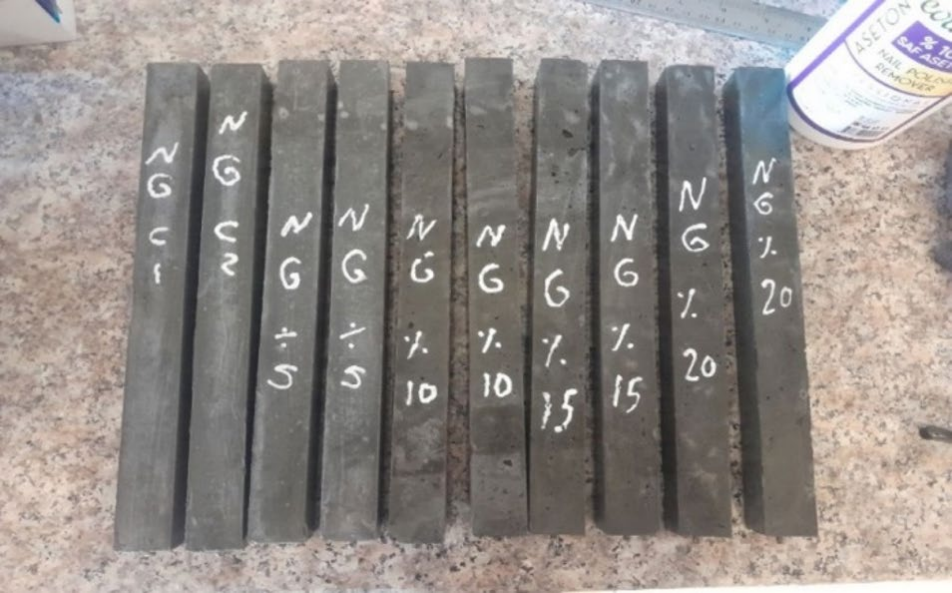
Remained samples for dry shrinkage test.

Dry shrinkage is one of the most important properties that is considered as required for knowing the effect of the added material to modify the mix on expansion and shrinkage properties of the produced mix. Based on the given detail and procedure in ASTM, C596^81^, the required samples for each mix have been prepared, which have dimensions of 2.5 × 2.5 × 28.5 cm, as shown in Fig. 11. After spending 24 h on sample preparations, the mixes that contain 30, 40, and 50% of nano glasses have shrunk, and the prepared samples have been broken, as shown in Fig. 12. For that reason, only the other mix samples have been brought out, and their readings have been recorded as shown in Fig. 13.

#### Water absorption, density, volume of permeable porous

**Fig. 14:**
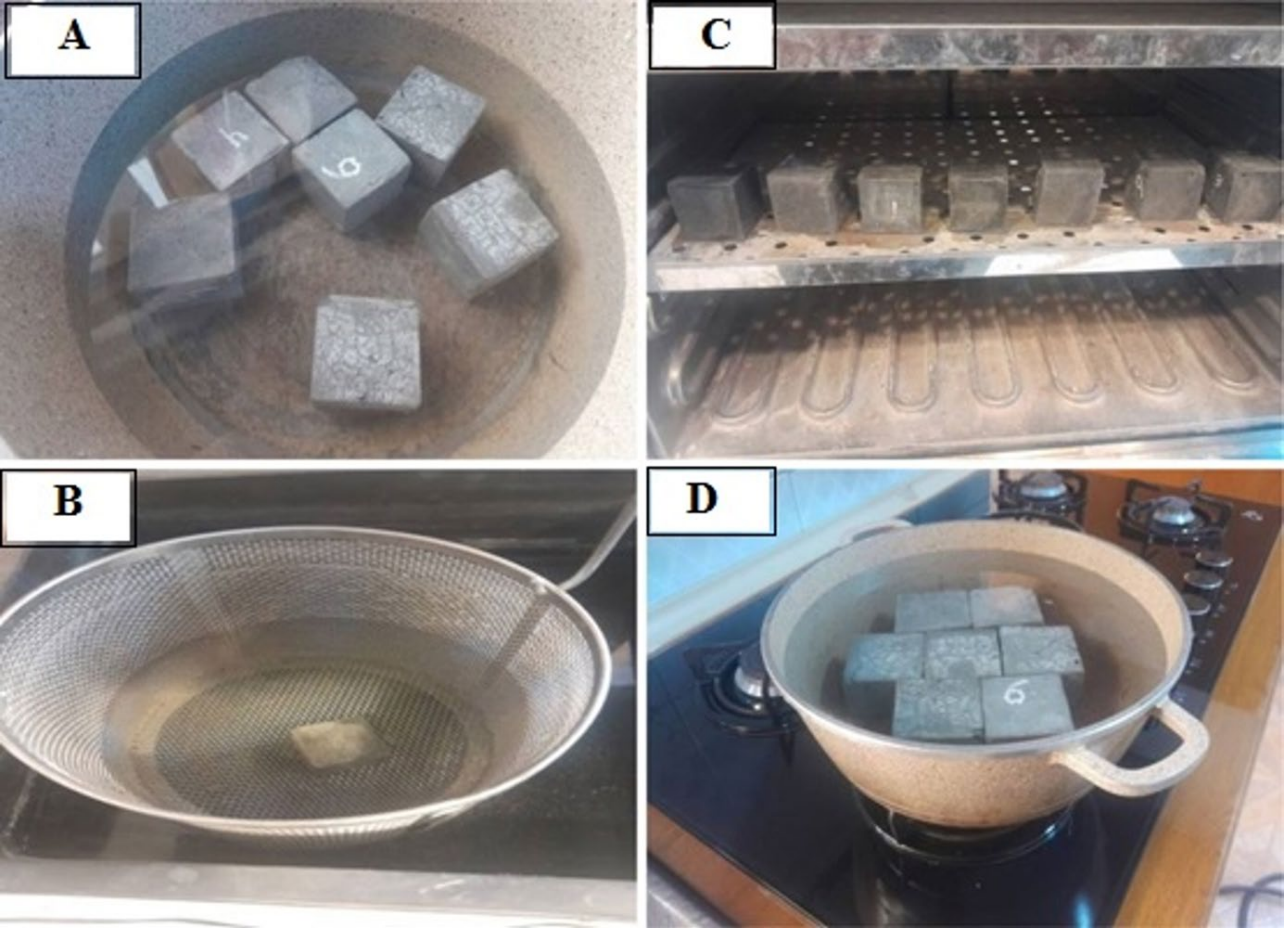
**a** Prepared samples in water bath. **b** Samples in apparent state in water bath, **c** Samples in oven. **d** Samples in boiling state.

As expressed in the ASTM, C 642^82^, for each mix, three samples have been prepared and cured as shown in Fig. 14a, After the specific curing time, thee samples weight have been recorded as saturated weight, samples have been putted in the basket submerged in water as shown in Fig. 14b, to find their apparent weights. Samples have been put in the oven for 24 h, as shown in Fig. 14c. After time passed, samples were brought out and their weights were recorded as dry weight. Finally, samples have been put in the water contained and boiled for 14 h, as shown in Fig. 14d, to find the sample’s weight after the boiling process. With having these data for each mix, and with using the given equations in the above specification sections, the density, water absorption, and volume of permeable porous material in each mix have been found, which show the effect of added material to each mix on these properties compared to the control mix.

## Result and discussions

After testing all the prepared samples, the following results have been obtained as expressed in the followed graphs and sub sections;

### Flow

**Fig. 15:**
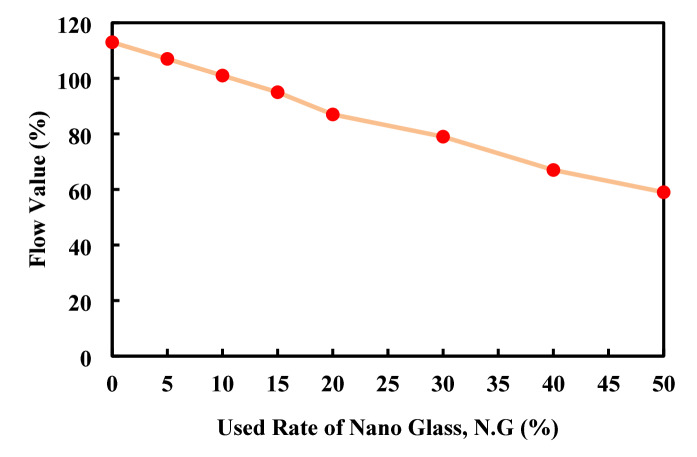
Flow value based on the used rate of nano-glass.

Figure 15 shows that increasing nano glass replacement in cement paste consistently reduces flow values. The control mix (0%) has the highest flow at 113%, which steadily drops to 87% at 20% replacement. Beyond 20%, flow declines more sharply, reaching 59% at 50% replacement. This decrease is due to nano glass’s fine particles increasing water demand and reducing workability. Up to 20%, the flow reduction is moderate, but above 30%, it becomes significant and may require admixtures or water adjustments. Overall, the data indicates a near-linear to slightly exponential drop in flow, emphasizing the need to balance nano glass benefits with workability challenges. Due to the un-availability of the nano glass in the previous work, the obtained result has been compared to the other material which is waste lass but with powder size. Elaqra and Rustom^61^, approved that when used waste lass powder as partial replacement of cement in paste with five different rate of the glass powder including 0, 10, 20, 25, and 30%, decrease the flow value for both type of glass powder. The obtained result by Ziejewska et al.^83^, supported the obtained result in this article since with the use of the waste lass powder with four different rate including 0, 10, 20, and 30% in geo-polymer composition decrease the flow of the produced mix with the increase of the used rate of lass powder.

### Compressive strength

**Fig. 16:**
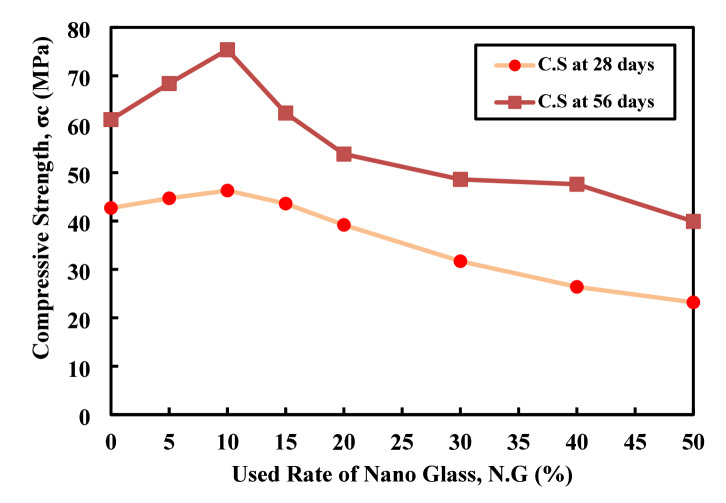
Effect of nano glass replacement on C.S of paste at 28 and 56 Days.

The experimental data show the influence of nano glass replacement on C.S over (28, 56) days as shown in the Fig. 16. At 28 days, C.S increases from 42.7 MPa (0% replacement) to a peak of 46.3 MPa at 10% nano glass, indicating that limited replacement enhances early strength likely due to the filler effect and pozzolanic activity of finely divided glass particles. Beyond 10%, the strength declines steadily, reaching only 23.2 MPa at 50% replacement, suggesting that excessive nano glass reduces the available cementitious material and impairs matrix cohesion. A similar trend appears at 56 days, where strength rises markedly to 75.38 MPa at 10% replacement about 24% higher than the control before decreasing progressively with higher replacement rates. Notably, all mixes show higher C.S at 56 days compared to 28 days, confirming the continued pozzolanic reaction over time. However, this long-term gain is not sufficient to offset the strength loss at high replacement levels. Overall, incorporating up to 10% nano glass improves both early and later-age strength, while replacement rates beyond 15–20% significantly compromise mechanical performance. Patel et al.^62^, used two type of waste lass powder the first one has 75 micro meter size as partial replacement of cement with five different rate including 0, 5, 10, 15, and 20%, the compressive strength has been investigated with three different curing time 7, 28, and 90 days at the all curing time the compressive strength was continuous to be decrease, while the size of the used waste lass powder has been changed to 63 micro the decrease rate in the compressive strength was lower and by receiving to 90 days the compressive strength of the modified mix are as control mx which indicate that the size of the waste granular provide significant role in compressive strength value.

### Flexural strength

**Fig. 17:**
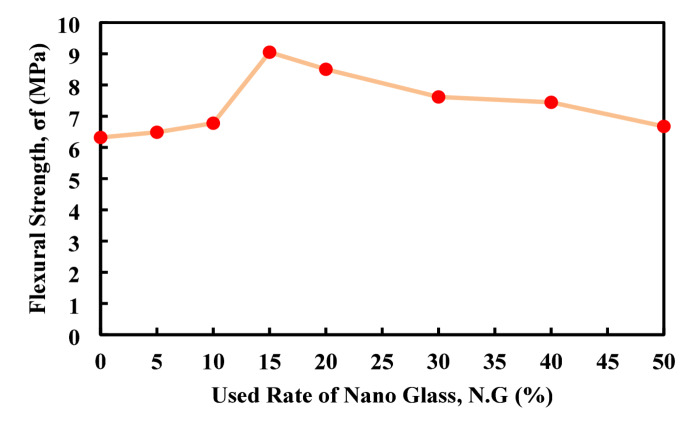
Flexural strength value based on the used rate of nano-glass.

The experimental data on flexural strength at 28 days, as shown in Fig. 17, indicate a clear enhancement in performance with the incorporation of nano glass up to an optimal level. Starting from a baseline of 6.32 MPa at 0% replacement, the flexural strength increases gradually, reaching 6.78 MPa at 10%, and peaks significantly at 15% replacement with a value of 9.05 MPa (about 43%) higher than the control. This suggests that nano glass positively contributes to matrix densification and crack-bridging properties, especially at moderate dosages. However, beyond 15%, the flexural strength begins to decline, dropping to 6.67 MPa at 50%, though still slightly above the control. The decline at higher replacement rates may be attributed to a reduction in cementitious content and weaker bonding within the matrix. Overall, nano glass improves the flexural strength up to 15–20% replacement, with 15% providing the optimal balance between strength gain and material substitution, while excessive replacement reduces the effectiveness of the mix. Nahi et al.^64^ investigate the usage of glass powder as a partial replacement of cement in the paste and mortar with five different rates (0, 10, 25, 35, and 60%), at the result of the compressive strength test with three different curing time (7, 28, and 90) using glass powder up to 10% provide higher value of the compressive strength compare to the control mix with other modified mixes which support the obtained result with the usage of the nano glass.

### Durability

**Fig. 18:**
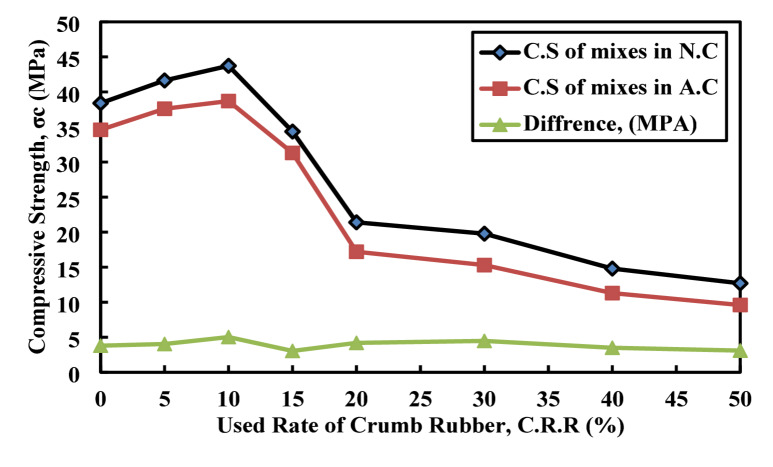
Effect of nano glass replacement on compressive strength of paste before and after acid attack.

Figure 18 illustrates the C.S behavior of paste specimens subjected to acid attack across various nano glass replacement levels (0% to 50%). The results show that C.S after acid attack initially increases from 34.6 MPa at 0% to a peak of 38.7 MPa at 10% replacement, indicating improved acid resistance with nano glass addition up to this level. However, beyond 10%, a noticeable decline in strength is observed, dropping sharply to 9.6 MPa at 50% replacement. A similar pattern is evident in the C.S with acid attack (prior to degradation), which also peaks at 43.73 MPa at 10% and then decreases steadily with higher replacement rates. The difference between C.S before and after acid attack ranges from 3.05 to 5.03 MPa, reflecting a relatively consistent degradation rate. Nonetheless, the degradation is slightly more pronounced at higher replacement levels, particularly at 40% and 50%. Overall, the results suggest that nano glass incorporation enhances acid resistance up to an optimal 10% replacement level, beyond which the durability of the material decreases, possibly due to brittleness or poor dispersion at higher nano glass contents. Kamali and Ghahremaninezhad^11^ investigate the effect of the usage of glass powder as the partial replacement of cement on the durability in the form of the chloride penetration, which obtained that with the increase in the usage of waste glass powder decrease the energy passage through the paste sample which indicate the reduction in the void content.

### Thermal conductivity

**Fig. 19:**
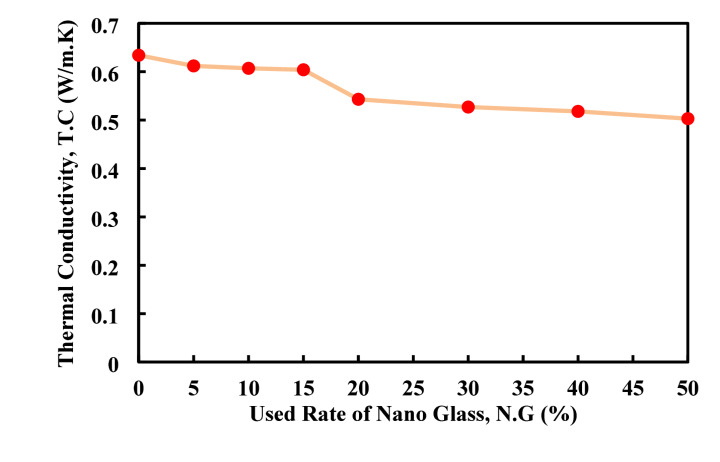
Effect of nano glass usage on thermal conductivity of the cement paste.

Figure 19 illustrates the effect of nano glass replacement on the thermal conductivity of the paste. The data indicate that increasing the nano glass content consistently reduces thermal conductivity, starting from 0.634 W/m K at 0% replacement and decreasing gradually to 0.503 W/m.K at 50% replacement a reduction of approximately 20.7% compared to the control. The most significant decline occurs between 15 and 20%, where thermal conductivity drops from 0.604 to 0.543 W/m.K. This trend suggests that nano glass serves as an effective thermal insulator, likely due to its lower intrinsic thermal conductivity and its ability to refine the pore structure, which reduces heat transfer pathways within the matrix. Overall, incorporating nano glass improves the composite’s thermal insulation, with replacement levels above 20% being especially beneficial for applications demanding enhanced thermal performance without greatly compromising mechanical properties. Same result has been obtained by Du et al.^84^, when used waste glass powder as partial replacement of paste with six different rate including 0, 5, 10, 15, 20, and 25% decrease the value of the thermal conductivity from 0.92 W/m K in control mix to 0.74 W/m K when 25% of the glass powder has been used as partial replacement of cement in paste.

### Fire resistance

**Fig. 20:**
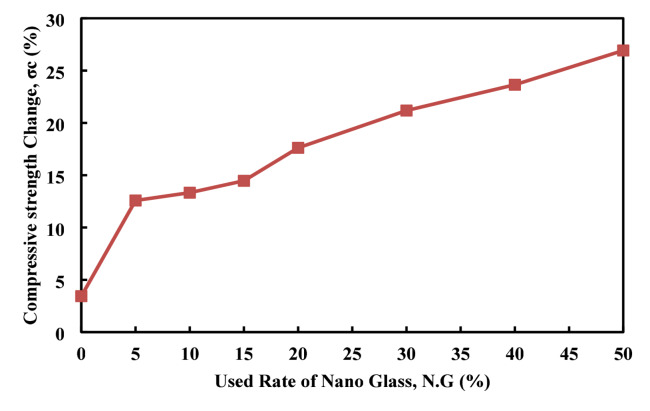
Effect of nano glass replacement on compressive strength changes of cement paste after fire exposure.

**Fig. 21:**
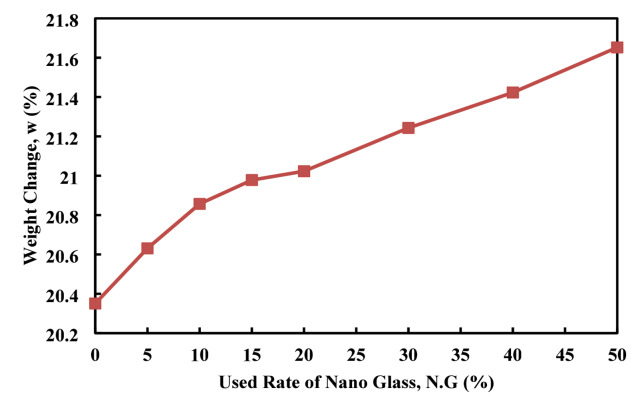
Effect of nano glass replacement on weight changes of cement paste after fire exposure.

Figures 20, 21 shows the effects of fire exposure on C.S and weight changes in cement paste with varying nano glass replacement rates. The experimental data show that C.S changes (%) increase progressively with higher nano glass content, starting from a minimal 3.44 at 0% replacement and rising significantly to 26.92% at 50% replacement. This indicates that nano glass incorporation enhances the cement paste’s ability to retain or possibly improve strength after fire exposure, likely due to improved microstructure and thermal stability. In contrast, weight changes (%) follow the change in continuous rate starting from 20.35% at 0% replacement which become 21.652 at 50%. This weight loss is attributed to moisture evaporation and decomposition of hydration products during fire exposure. The higher weight change observed at 50% replacement may show increased porosity or material degradation at excessive nano glass content. Overall, the data showed that increasing nano glass replacement enhances post-fire C.S retention while moderately affecting weight loss, with an optimal balance likely between 20 and 30% replacement for improved fire resistance and stability. Adversely to the obtained result by Ziejewska et al.^83^, who obtained that the usage of the glass granular can cause the improve heat resistance by remaining the mass of the structure and decrease in the weight losses rate.

### Water absorption, density, and volume of permeable porous

**Fig. 22:**
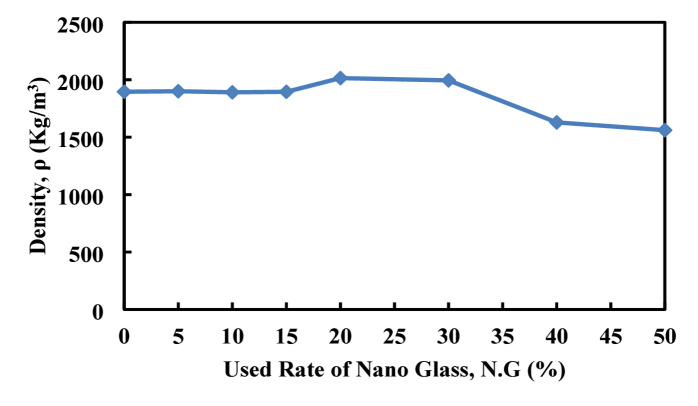
Effect of nano glass replacement on paste density.

**Fig. 23:**
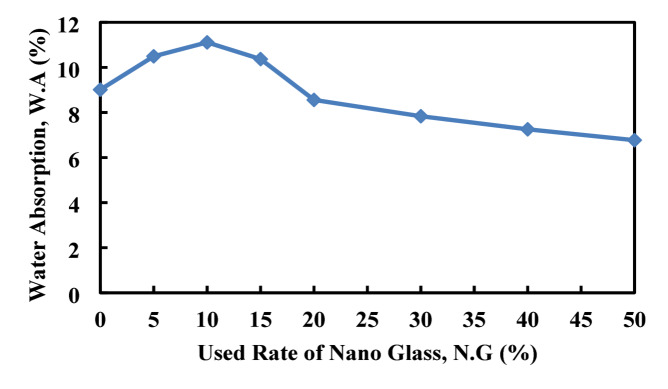
Effect of nano glass replacement on paste water absorption.

**Fig. 24:**
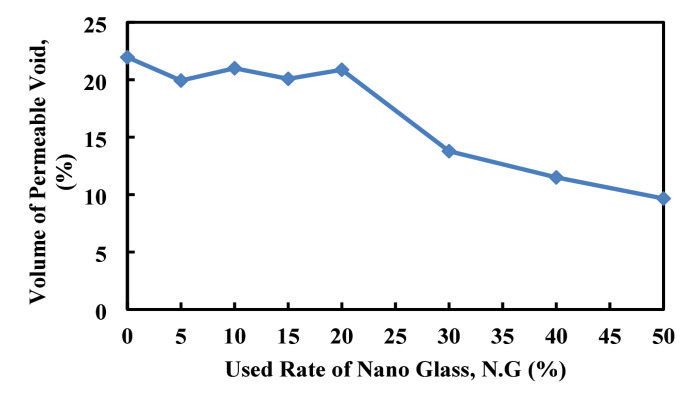
Effect of nano glass replacement on paste permeable voids.

Figures 22, 23, 24, illustrates the effect of nano glass replacement on paste’s density, water absorption, and permeable voids. Up to 20% replacement, density remains stable around 1890–1900 kg/m^3^, with a slight increase at 20% (2014.5 kg/m^3^), indicating a densification effect. However, at 50% replacement, density drops sharply to 1560.97 kg/m^3^, likely due to the lower specific gravity of nano glass and reduced solid content. Water absorption initially increases, peaking at 11.1% at 10% replacement, indicating higher porosity or incomplete densification at low to moderate replacement levels. Afterward, it declines steadily, reaching 6.77% at 50%, suggesting that higher nano glass content improves the material’s resistance to water ingress, possibly by filling micro voids and refining the pore structure. Similarly, the volume of permeable voids decreases significantly with increasing nano glass content. Starting from 21.96% at 0% replacement, it drops to 9.66% at 50%. This reduction in voids reflects an improvement in the microstructural compactness and suggests better durability performance at higher replacement levels. Although lower replacement levels (up to 15%) may slightly increase water absorption and maintain similar density, higher replacement levels (30–50%) substantially reduce water absorption and permeable voids, enhancing the material’s resistance to moisture and potential durability. However, the sharp decrease in density at 50% could impact mechanical strength.

### Dry shrinkage

**Fig. 25:**
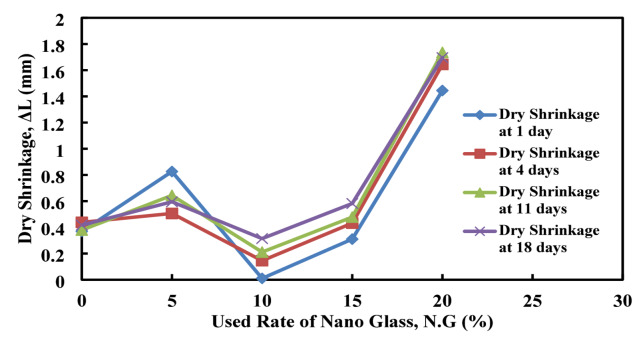
Effect of nano glass replacement on drying shrinkage over time.

As shown in Fig. 25, the data presents that drying shrinkage decreases at lower nano glass replacements (10–15%), with 10% showing the least shrinkage 0.01 mm at 1 day and 0.314 mm at 18 days indicating better dimensional stability. In contrast, 20% replacement causes the highest shrinkage, peaking at 1.736 mm by day 11, likely due to reduced cement cohesion. The control (0%) shows moderate shrinkage, while 5% and 15% have intermediate values. Overall, 10% nano glass is optimal for minimizing shrinkage, but higher amounts, especially 20%, increase shrinkage risks.

### X-ray diffraction test (XRD)

**Fig. 26:**
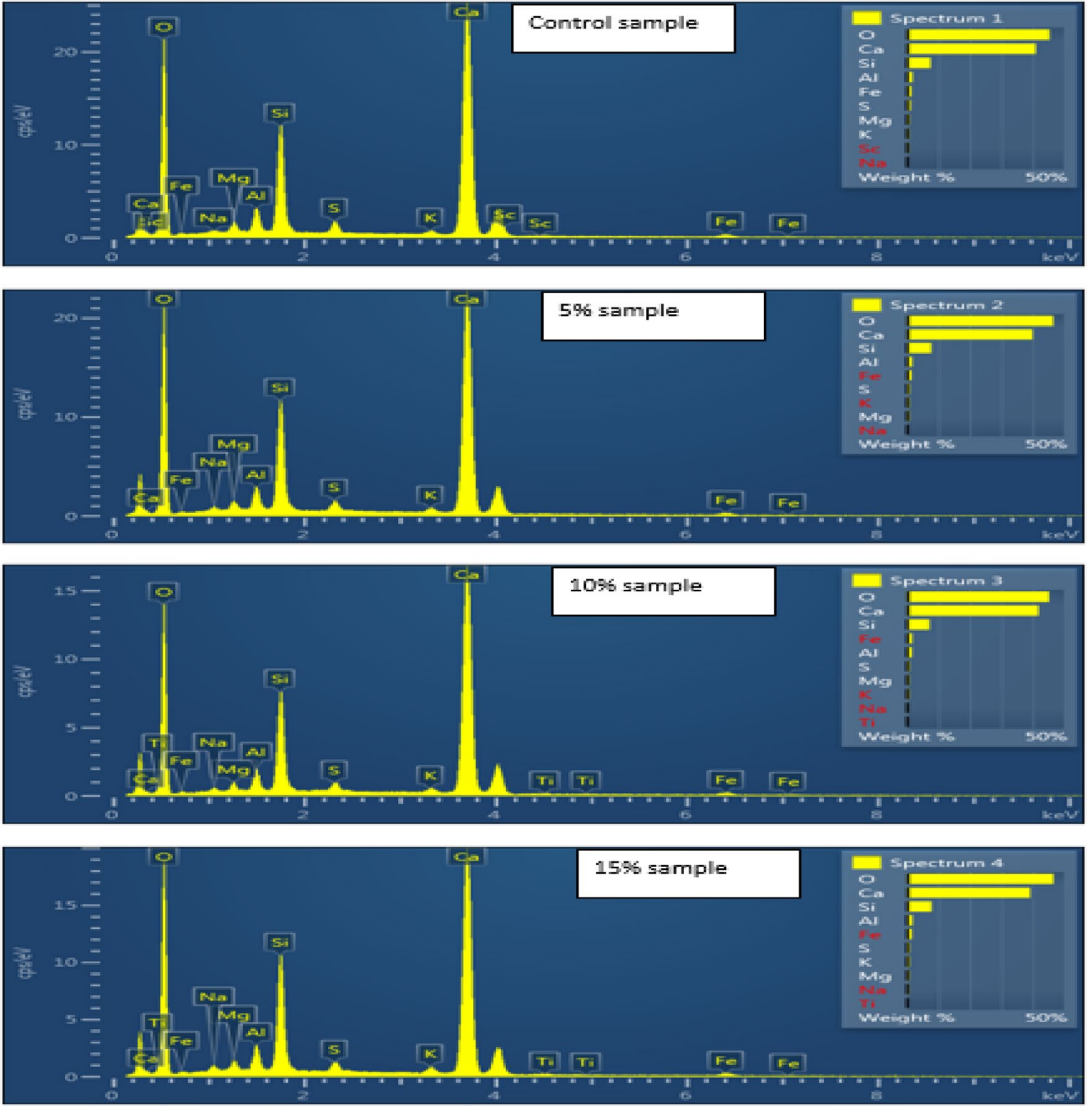
XRD elemental analysis of paste with varying nano glass replacement levels (control, 5%, 10%, 15%).

Table 4 and Fig. 26 show that XRD analysis of Control, 5%, 10%, and 15% nano glass samples reveals dominant oxygen and calcium levels, indicating a calcium silicate matrix like wollastonite (CaSiO_3_).The Control sample shows high oxygen (45.39 wt%) and calcium (41.07 wt%), with moderate silicon (7.54 wt%) and aluminum (1.69 wt%). Scandium (0.44 wt%) appears only in the Control, showing dilution or removal in modified mixes. In the 5% sample, oxygen rises (46.64 wt%) and calcium drops (40.03 wt%), with increased sodium (0.30 wt%), potassium (0.64 wt%), and silicon (7.81 wt%), indicating feldspar phases. The 10% sample shows calcium recovery (41.93 wt%) and a slight oxygen decrease (45.25 wt%). Titanium (0.19 wt%) appears, possibly introduced via accessory minerals, and iron reaches its highest level (1.63 wt%). At 15% replacement, oxygen rises to 46.71 wt%, while calcium decreases further to 39.27 wt%. This sample also shows the highest sodium (0.45 wt%) and potassium (0.81 wt%) levels, indicating a stronger presence of alkali-bearing minerals like K-feldspar. Aluminum remains relatively stable across all samples (~ 1.56–1.69 wt%), maintaining the alumino-silicate background. Overall, the transition from the Control to 15% replacement reflects a gradual shift from a calcium-silicate matrix to a more alkali- and trace-element-enriched composition. The loss of scandium and the introduction of titanium further emphasize mineralogical changes, with the 15% sample showing the greatest variation, which may influence the material’s performance.

**Table 4:** XRD elemental analysis.

Element	Line type	Apparent concentration	k ratio	Wt%	Wt% Sigma	Atomic %	Standard label	Factory standard
Control Sample								
O	K series	5.33	0.01793	46.64	0.31	66.86	SiO2	Yes
Na	K series	0.05	0.00023	0.30	0.06	0.30	Albite	Yes
Mg	K series	0.09	0.00061	0.60	0.05	0.57	MgO	Yes
Al	K series	0.26	0.00187	1.61	0.06	1.37	Al2O3	Yes
Si	K series	1.30	0.01033	7.81	0.11	6.38	SiO2	Yes
S	K series	0.15	0.00128	0.89	0.06	0.64	FeS2	Yes
K	K series	0.12	0.00100	0.64	0.06	0.37	KBr	Yes
Ca	K series	6.71	0.05992	40.03	0.27	22.91	Wollastonite	Yes
Fe	K series	0.20	0.00195	1.48	0.17	0.61	Fe	Yes
5% of nano glass replacement								
O	K series	5.33	0.01793	46.64	0.31	66.86	SiO2	Yes
Na	K series	0.05	0.00023	0.30	0.06	0.30	Albite	Yes
Mg	K series	0.09	0.00061	0.60	0.05	0.57	MgO	Yes
Al	K series	0.26	0.00187	1.61	0.06	1.37	Al2O3	Yes
Si	K series	1.30	0.01033	7.81	0.11	6.38	SiO2	Yes
S	K series	0.15	0.00128	0.89	0.06	0.64	FeS2	Yes
K	K series	0.12	0.00100	0.64	0.06	0.37	KBr	Yes
Ca	K series	6.71	0.05992	40.03	0.27	22.91	Wollastonite	Yes
Fe	K series	0.20	0.00195	1.48	0.17	0.61	Fe	Yes
10% of nano glass replacement								
O	K series	3.54	0.01190	45.25	0.33	65.80	SiO2	Yes
Na	K series	0.04	0.00017	0.31	0.06	0.32	Albite	Yes
Mg	K series	0.06	0.00043	0.60	0.05	0.57	MgO	Yes
Al	K series	0.18	0.00129	1.56	0.06	1.35	Al2O3	Yes
Si	K series	0.85	0.00673	7.19	0.11	5.95	SiO2	Yes
S	K series	0.09	0.00078	0.77	0.06	0.56	FeS2	Yes
K	K series	0.08	0.00064	0.57	0.06	0.34	KBr	Yes
Ca	K series	4.99	0.04458	41.93	0.30	24.34	Wollastonite	Yes
Ti	K series	0.02	0.00017	0.19	0.09	0.09	Ti	Yes
Fe	K series	0.15	0.00153	1.63	0.18	0.68	Fe	Yes
15% of nano glass replacement								
O	K series	4.85	0.01632	46.71	0.34	66.87	SiO2	Yes
Na	K series	0.07	0.00031	0.45	0.06	0.45	Albite	Yes
Mg	K series	0.09	0.00057	0.62	0.06	0.59	MgO	Yes
Al	K series	0.25	0.00178	1.69	0.07	1.44	Al2O3	Yes
Si	K series	1.18	0.00935	7.85	0.12	6.40	SiO2	Yes
S	K series	0.13	0.00110	0.85	0.06	0.61	FeS2	Yes
K	K series	0.14	0.00115	0.81	0.07	0.48	KBr	Yes
Ca	K series	5.93	0.05296	39.27	0.29	22.45	Wollastonite	Yes
Ti	K series	0.02	0.00020	0.17	0.09	0.08	Ti	Yes
Fe	K series	0.19	0.00187	1.57	0.18	0.65	Fe	Yes

### Scanning electron microscope test (SEM)

**Fig. 27:**
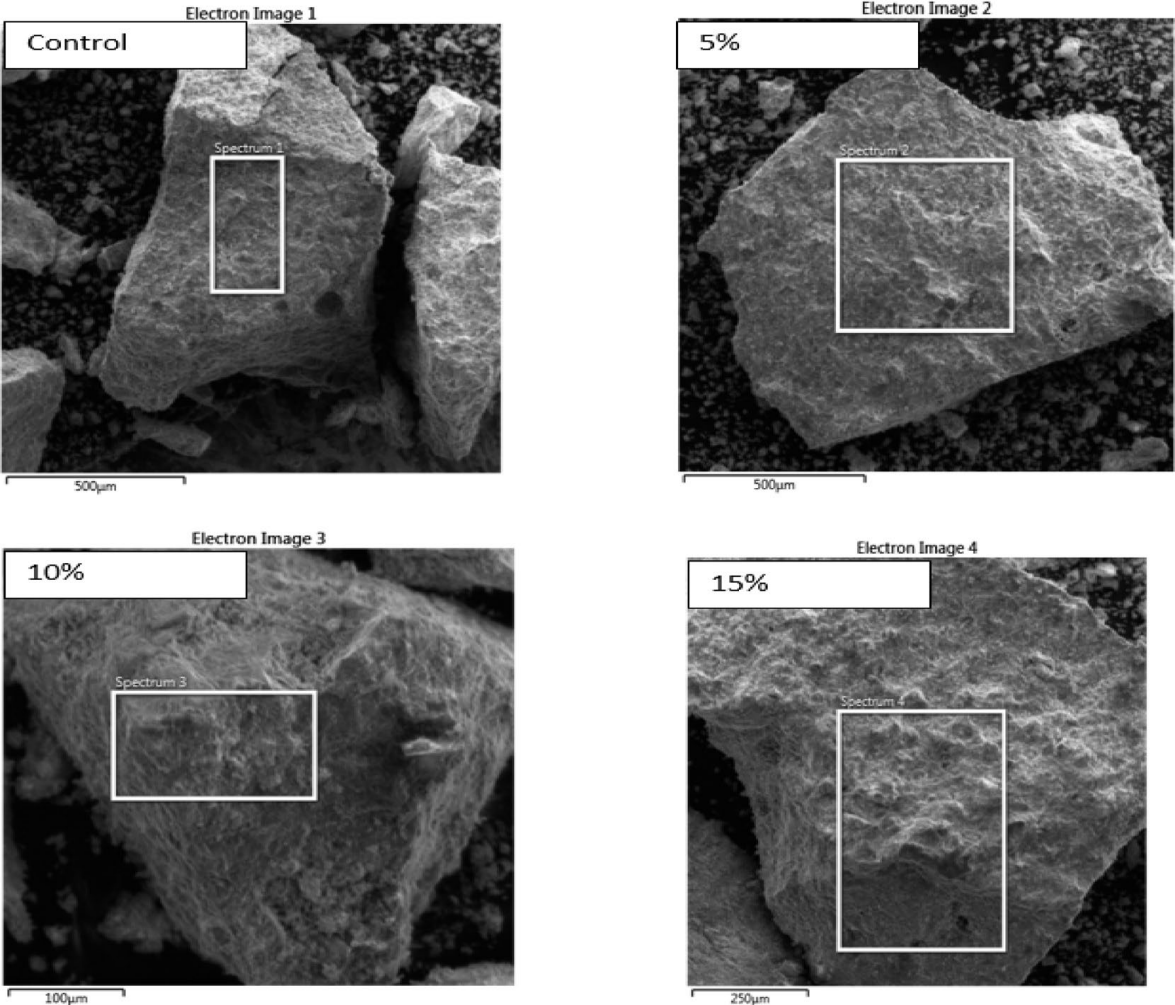
Influence of nano glass dosage on the microstructure and performance of cement paste.

As shown in Fig. 27, the effect of nano glass on cement paste is highly dosage-dependent. At lower replacement levels, nano glass primarily acts as a filler and provides nucleation sites for hydration products, leading to a denser microstructure and reduced porosity, which enhances early-age strength. As the dosage increases, its pozzolanic activity becomes more significant, reacting with calcium hydroxide to generate additional calcium silicate hydrate (C–S–H), thereby improving mechanical strength and durability. However, at higher dosages, excessive nano glass may cause particle agglomeration, negatively impacting workability and resulting in uneven dispersion. This can hinder uniform hydration and strength development. Therefore, while optimal amounts of nano glass improve the paste’s performance, excessive use may introduce challenges in mix consistency and overall quality.

## Conclusions

An experimental investigation was conducted to evaluate the effects of incorporating waste nano glass as a partial cement replacement in cement paste at eight different replacement levels on its fresh, mechanical, and durability properties. The main findings are summarized as follows:

1. Workability decreased progressively with increasing nano glass content, mainly due to its fine particle size and higher water demand, indicating that mix adjustments or the use of chemical admixtures may be required at higher replacement levels.
2. Compressive and flexural strengths improved at moderate nano glass replacement levels, reaching peak values at 10% and 15%, respectively. Beyond these thresholds, mechanical performance declined, primarily due to cement dilution and the development of microstructural discontinuities.
3. Enhanced durability was observed through increased resistance to acid attack, reduced thermal conductivity, and improved fire performance, particularly at moderate to high levels of nano glass replacement. The 10–20% range proved most effective in boosting these properties while maintaining structural integrity**.**
4. As nano glass content increased, particularly beyond 30%, water absorption and permeable porosity decreased, suggesting a more compact microstructure. However, very high replacement levels (40–50%) resulted in a noticeable drop in density, which may adversely impact overall strength.
5. Shrinkage reached its minimum at 10% nano glass replacement, whereas the highest shrinkage occurred at 20%, highlighting the critical need to carefully manage the replacement dosage, Since the using of the waste glass higher than this rate cause the crack in the paste due to the high amount of shrinkage.
6. XRD and SEM analyses revealed that adding nano glass led to changes in microstructure and composition, including higher alkali levels, lower porosity, and greater formation of hydration products corroborating the observed enhancements in material performance.
7. Overall, nano glass powder demonstrates significant potential as a partial cement replacement, providing notable improvements in strength, durability, and thermal performance when used at moderate dosages. An optimal replacement ranges of 10–20% achieves a balanced enhancement of mechanical properties, durability, and workability.
8. In contrast, higher replacement levels (≥ 30–40%) adversely affect performance due to reduced matrix cohesion, increased porosity, and diminished workability.
9. These findings confirm nano glass as a sustainable and eco-friendly additive capable of improving cementitious materials while promoting waste recycling.

## Limitation of the manuscript

This study is subject to several limitations. First, the nano glass was produced from crushed waste bottle glass; variations in glass type and chemical composition may lead to different hydration behavior and performance outcomes. Second, the experiments were conducted using Ordinary Portland Cement from a single manufacturer; cements from other sources or with different chemical compositions may interact differently with Nano glass. Finally, tap water was used for mixing, and changes in water chemistry could influence hydration reactions and measured properties. These factors should be considered when generalizing the findings, and further studies are recommended to evaluate the effects of material variability.

## Data Availability

All data generated or analyzed during this study are included in this published article.
